# Health Challenges Among Waste Collectors in Bangladesh: Exploring Risk Factors Using Multi-level Modeling

**DOI:** 10.1016/j.shaw.2024.10.007

**Published:** 2024-11-14

**Authors:** Safayet Hossain, Md Farhad Hossain, Bowen Liu, Anjuman Ara, Haneen Alsaoud, Md Abdul Majed Patwary

**Affiliations:** 1 Department of Statistics, Comilla University, Cumilla 3506, Bangladesh; 2 Department of Mathematics and Statistics, Division of Computing, Analytics and Mathematics, Science and Engineering, University of Missouri, Kansas-City, 64110, USA; 3 Department of Chemistry, Comilla University, Cumilla 3506, Bangladesh

**Keywords:** Diseases, Machine learning, Multi-level modeling, Occupational health, Waste collectors

## Abstract

**Background:**

Waste collectors face multi-faceted health risks including exposure to musculoskeletal disorders, respiratory diseases, and workplace injuries because of their hazardous work environment. The purpose of this study was to determine the risk factors that affect health of the waste collectors across Bangladesh.

**Methods:**

Data on a cross-sectional survey obtained information from 481 waste collectors about their demographics, housing conditions, hygiene practices, security measures, and disease prevalence. Descriptive analyses and multi-level models are used.

**Results:**

As per univariate analysis, 81.3% did not have access to clean water, 58.4% did not have access to sanitation, and 65.9% of people lived in unhealthy housing. According to bivariate analysis, there is an association between unhealthy working conditions i.e., respiratory illnesses (19.1% asthma, 29.7% cough), gastrointestinal issues (59.3% discomfort, 24.1% diarrhea), musculoskeletal disorders, and dermatological diseases, and unsafe working conditions (i.e., 60% no masks, 71% no gloves, and 75% no boots). Considering geographic clustering, multi-level modeling examined how different factors affected particular illnesses. The following were significant protective factors: better housing (80% lower odds of acute irritation), security measures (50% lower odds of gastrointestinal diseases), good hygiene (62% lower odds of dermatological diseases), and younger age (2% higher odds of respiratory diseases per year).

**Conclusion:**

In summary, unsafe living and working conditions greatly push the risks of illness for waste collectors. These risks to occupational health can be reduced with targeted interventions that enhance housing, hygiene, security protocols, and working conditions.

## 1 Introduction

Household garbage collectors are susceptible to several health risks, such as exposure to bioaerosol, musculoskeletal disorders, skin conditions, respiratory diseases, workplace injuries, mental health disorders, work-related stress, and burnout [[1], [2], [3]]. Trash collection for homes may be hazardous and require a lot of work [4,5]. It entails doing maintenance on a car that is driven in all weather conditions [6]. These collectors are more vulnerable to microbial exposure because of changes in waste management procedures. This might lead to health issues such as declining respiratory function, irritated eyes and skin, and digestive issues [7]. They also handle physical health issues, including musculoskeletal problems, skin conditions, and respiratory infections, which are more prevalent in those who have worked for a long time [8,9]. Among garbage collectors, who frequently lack access to health insurance and suitable occupational safety methods are occupational injuries, psychological problems, work-related stress, and burnout. Furthermore, work pace and rhythms are thought to be important factors in home garbage collection efficiency. Injuries in HWCs are frequent due to these causes [10].

Waste collectors in Bangladesh are primarily untrained laborers who frequently face discrimination and exclusion from social services. This is particularly valid in urban settings. They labor long hours in difficult circumstances handling hazardous chemicals and combined trash without proper tools or training [11]. Their health and well-being are seriously jeopardized by this physical strain, exposure to hazardous substances, and unhygienic surroundings [12]. Surprisingly, new data indicate that garbage collectors are seeing an alarming rise in health-related issues, such as skin diseases, respiratory ailments, and accidents sustained while working their hazardous occupations [13]. According to Ahmed (2019), Bangladesh’s urban areas produce about 25,000 tons of solid waste per day or 170 kg per person annually [14,15]. According to research conducted between 2018 and 2020, waste collectors in Dhaka, Bangladesh, have a 40% increased risk of respiratory infections as well as higher incidences of skin diseases, musculoskeletal illnesses, and mental health difficulties. Improving working conditions, putting in place suitable safety precautions, and implementing health programs are necessary to address these issues [16]. The volume of garbage has increased every 15 years during the past three decades. In urban areas, the collection efficiency varies from 37% to 77%, with an average of 55% of solid waste being uncollected [17].

The infections and risk factors associated with solid waste management among waste disposal workers in Bangladesh have been the subject of numerous studies in this field [18]. Research on model development, however, is lacking. Another study focused on the health, occupational risks, and socioeconomic characteristics of informal waste workers [19]. A comparative study between waste pickers and non-waste pickers concentrated on examining health problems faced by child waste collectors [20]. The occupational health risks that child waste collectors in South Asia undergo are examined in this scoping review [21]. Previous studies have all attempted to investigate the risk factors related to garbage collectors’ health.

Various approaches have been employed in previous research projects to gather data, including expert interviews and personal observations, face-to-face questionnaire survey, 24-hour recall method, body mass index (BMI) and BMI for age Z-score, and mixed methodology approach [11,22]. The data analysis was performed using multiple techniques including descriptive, bivariate, inductive content analysis approach, analysis of socioeconomic status, hygiene practices, and microbial exposure, and exploratory analysis. This research aims to explore the risk factors of waste collectors all over Bangladesh. While earlier research attempted to pinpoint the risk factors that influence waste collectors’ health, was very limited. Furthermore, every earlier study focused on a certain area. By attempting to examine the study population across Bangladesh, this study attempts to fill this gap.

This research investigates the risk factors faced by waste collectors throughout Bangladesh. Prior research tried to identify the specific components that affect the health of garbage collectors, but its scope was severely restricted. Moreover, the study area was limited. No attention has been dedicated to this specific demographic nationwide. This study aims to address the gap by collecting data from six out of the eleven major municipal corporations in Bangladesh. This study is characterized by its distinctiveness. Furthermore, we have compiled data on 24 distinct categories of illnesses, encompassing respiratory, dermatological, musculoskeletal, gastrointestinal, acute irritation, and mental health conditions, which are prevalent among garbage collectors across the globe. By determining the prevalence of diseases, it is possible to gain a clear understanding of the general health status of garbage collectors. This study aims to address the methodological gap by employing multi-level regression analysis, which allows for the examination of regional differences in the health status of garbage collectors. It will assist the government and policymakers in identifying the issues and formulating policies regarding this group. Furthermore, it focuses on identifying the impact of various factors on various diseases using a multi-level model by observing random effects area-wise.

## 2 Materials and methods

### 2.1 Sampling and data collection

Waste collectors across the 11 city corporations in Bangladesh are targeted. The registered trash collectors are taken into consideration. It is a cross-sectional study. This study uses primary data from a 43-question closed-ended questionnaire given to interviewers. The study area was selected by simple random sampling and stratified random sampling. Six city corporations were chosen from an initial pool of eleven city corporations using simple random sampling. Then using stratified random sampling, wards from each city corporation were chosen for the second stage. Next, a random selection of participants was gathered from these wards. A random sample of 481 garbage collectors is chosen (Supplementary Fig.1). The sample size is determined based on the Cochran’s method. It is a reliable and suggested method widely used in quantitative research. The response rate was 100%. The survey took place in six main Bangladeshi city corporations: Dhaka, the capital; Chittagong, the commercial capital; Cumilla, Narayanganj, Khulna, and Barisal. The questionnaire covers facilities, personal hygiene, safety, and disease-related symptoms like respiratory, skin, gastrointestinal, acute irritation, musculoskeletal, dermatological, and psychological. This study utilizes the Kobo toolbox to collect primary data using an organized questionnaire.

#### 2.1.1 Ethical statement

This study employed a primary dataset, and participants were not given any monetary incentives to guarantee the reliability and confidentiality of the gathered data. At first, ethical approval was taken from the ethics committee. The IRB approval ID is ERC-2023-3028. Participants were notified of their entitlement to withdraw from the survey at any point without the requirement of providing a rationale. Prior to data collection, the participants were presented with the whole questionnaire and their agreement was obtained. In addition, the participants gave their approval for the publication of the analyzed survey findings, under the condition that their identifiable information would be excluded. The survey was carried out in person, strictly following the guidelines outlined in the Helsinki Declaration for research involving human participants.

### 2.2 Variables of interest

Sociodemographic variables like residence types, location, unhealthy residence, clean water, sanitary facilities, personal hygiene, and safety are independent variables. The personal hygiene index is calculated from hygiene activities like soap baths, baths after work, baths after defection, clean clothes, nails, etc. The safety index is calculated from safety instruments using simple summation. The mean technique was used to calculate the index of respiratory, dermatological, mental health, musculoskeletal, acute irritation symptoms, and gastrointestinal illnesses.

This study examines several outcomes. In the multi-level model, overall health status, respiratory diseases, dermatological diseases, mental health, musculoskeletal disorders, gastrointestinal diseases, and acute irritant symptoms were dependent variables. The outcome variables were not directly mentioned in the questionnaire. They were computed later by using the averaging method. Respiratory diseases, dermatological diseases, mental health, musculoskeletal diseases, acute irritation symptoms, and gastrointestinal diseases index were computed based on their respective diseases (Supplementary Table 11). For example, three diseases are considered under respiratory diseases: asthma, cough, and bronchitis. The respondents were asked whether they have suffered from these diseases in the last 6 months with the response “yes” or “no”. Then based on the presence or absence, the overall outcome variable of the respiratory diseases index was obtained using the averaging method (Supplementary Fig. 2). Similarly, dermatological disease, mental health, musculoskeletal diseases, acute irritation symptoms, and gastrointestinal diseases were calculated. Then overall health status was also computed from these outcome variables.

### 2.3 Statistical analysis

The results provide descriptions of response patterns for each variable. The proportion was calculated for all diseases, safety instruments, hygiene, and sociodemographic variables. A multi-level regression model elucidated the association between disease prevalence and covariates. This model consists of two components: random effect and fixed effect. A random effects model was used to analyze the variation in City Corporation in Bangladesh. The fixed effect model quantified the magnitude of the relationships between dependent variables and covariates. The study computed odds ratios (ORs) and 95% confidence intervals (CIs) for the outcome variable, using independent variables as predictors.

In multi-level linear mixed model, several key variables are controlled to address the confounding effects on the relationship between predictors and outcome variables. The study controlled for city corporations, which includes six cities, to capture geographic variability in health outcomes. Additionally, residential conditions and hygiene and safety maintenance were incorporated, accommodating their roles as both predictors and potential confounders. These adjustments allow isolating the specific contributions of these covariates on outcome variables.

### 2.4 Multi-level model

Multi-level mixed-effects models are powerful statistical models, also known as hierarchical linear models or random effects models, that expand linear regression to handle data with nested or clustered structures. It incorporates random and fixed effects [23]. Under many real-data scenarios, the response variables are binary. Then, a logit link function is used to model the response variables with the structure of a multi-level linear model. The parameter estimation of a multi-level linear model is usually addressed by utilizing a maximum likelihood or restricted maximum likelihood approach with the assumption of Multi-level model and the binomial logit function is used in this case.

(1) $$Logit\left(p\right)=\mathrm{Log}\left(\frac{p}{1-p}\right)=\beta_{0}+\beta_{1}x_{1}+...+\beta_{k}x_{k}+u_{i}$$

The model equation in this context is given below.

(2) $$\log\;it\left(p_{ij}\right)=\beta_{0}+\beta_{1}X_{1ij}+\beta_{2}X_{2ij}+...+\beta_{k}X_{kij}+u_{0i}+u_{1i}X_{1ij}+...+u_{ki}X_{kij}+e_{ij}$$

In a statistical model, the parameters that are regarded as constant at every data level are represented by fixed effects such as, $\beta_{0},\beta_{1}$. The average influence of factors on the result is frequently modeled using these effects. The general formula for the fixed effect model is

(3) $$Y_{ij}=\beta_{0}+\beta_{1}x_{1ij}+...+\beta_{k}x_{kij}+\varepsilon_{ij}$$

Conversely, variability that is specific to certain groups or data levels and cannot be explained by fixed effects is captured by random effects. It is presumed that these effects are taken from a distribution that illustrates the variation across various individuals or categories.

(4) $$Y_{ij}=\beta_{0}+u_{0i}+\beta_{1}x_{1ij}+u_{1i}x_{1ij}+...+\beta_{k}x_{kij}+u_{ki}x_{kij}+\varepsilon_{ij}$$

### 2.5 Evaluation process

Model validation includes residual plots to determine heteroscedasticity. Q-Q plots show if a dataset matches a theoretical distribution. The dharma (Diagnostics for Hierarchical Regression Models) also evaluates mixed-effects or hierarchical models’ residual homogeneity assumption. R’s model validation tool checks dispersion and visualizes residuals [24]. Regression analysis uses the variance inflation factor to measure multicollinearity between predictor variables [25,26].

## 3 Results description

### 3.1 Descriptive analysis

According to Supplementary Table 1, many Bangladeshi garbage removal personnel are poor and lack adequate water and sanitation. Due to the disparity of these qualities among municipal corporations, distinct techniques are needed. Data illustrates citywide harmful living inequalities. Khulna (84.8%), Cumilla (82.1%), and Chittagong (81.1%) have higher rates than Dhaka (25%). All city corporations had 65.9% garbage collectors living in unsanitary dwellings, showing a countrywide problem in Bangladesh. Thus, every city corporation may have gender-specific challenges. About 80% of the responses are male and 20% female. Types of dwellings are distributed differently. In Dhaka, 93.1% of garbage collectors live in buildings, unlike in nearby cities. Tin sheds (55.1%) dominate City corporation (CC) housing. Cumilla has 56.8% tin sheds, Khulna has 90.9%. The places where CC lives vary. Dhaka is 100% urban, unlike other cities that favor rural living. Respondents dwelling in cities are at 69.2%, municipalities at 12.3%, and villages at 18.5%. Additionally, sanitation and water access are inconsistent. Access is poor in Barisal and Cumilla. Clean water and sanitation are scarce in 58.4% and 81.3% of CCs. As in other cities, 89% of Cumilla and 93% of Khulna lack clean water. The data demonstrates that waste collectors' living and economic conditions differ substantially across city corporations.

Supplementary Table 2 shows hygiene of the respondents at 59% of the garbage collectors solid hazardous work factor versus 41%. The rate is low. The defecting respondents do not shower 27% of the time. Nearly 32% do not bath with soap. The most worrying finding was that over 48% of the subjects had dirty nails, a sign of various bacterial and viral diseases. In addition, 47% and 49% of the individuals reported dirty hair and clothing. The respondents said their occupations were unhealthy, tiring, and repetitious since they had to do hard work.

Supplementary Table 3 displays Bangladeshi garbage collectors’ protective gear. The data suggest that over 60% of garbage collectors do not use masks. Most respondents said they do not wear masks since they are used to it. About 71% of the respondents said they throw away trash without gloves. According to 75% of the respondents, they never wore work boots. Boots are exclusively used during defections. Approximately 52% do not wear an apron as previously noted. Aprons were used for identity, not safety, according to respondents. Financial constraints contribute to safety product underutilization because they are not always available. The data indicate that faulty safety measures cause several diseases.

Supplementary Table 4 illustrates vast disparities in disease prevalence among Bangladeshi municipal corporations. Cough prevalence is notable, with Chittagong having the most respondents (47.3%). This is higher than in Barisal, where 16.4% of respondents had a cough. With 37.1% of the respondents coughing, Dhaka is in the middle.

**Fig. 1:**
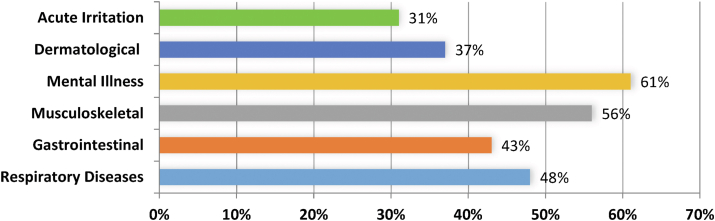
Types of diseases among the waste collectors.

Fig. 1 depicts the respondents’ ailments. About 48% of trash collectors have respiratory illnesses. In addition, 43%, 31%, 56%, 61%, and 37% of persons have gastrointestinal, acute irritation, musculoskeletal, mental illness, and skin issues. The data show that Bangladeshi waste collectors are plagued by infections.

This shows that cough may be linked to urbanization, higher population densities, and urban environmental factors. Overall, 29.7% of city corporation respondents coughed in the past six months. Khulna has the highest asthma rate (31.8%) and Narayanganj has the lowest (4.3%). Bangladesh’s asthma prevalence is 20%, while Chittagong’s 25.7% frequency stands high. Compared with Barisal (36.1%), Chittagong (18.9%) has a higher bronchitis rate. Overall, most city corporation respondents do not experience bronchitis, especially in Narayanganj (95.7%) and Chittagong (81.1%).

Beyond respiration, nausea and stomach ailments vary. Khulna had 48.5% of nausea respondents and Dhaka had 77.6% of gastrointestinal problems. An astonishing 52.7% of Chittagong residents have digestive issues. Diarrhea, anorexia, ophthalmic problems, and hyposmia vary regionally. The highest incidence of diarrhea (36.5%) and eye problems (9.5%) occur in Chittagong. The highest hyposmia rate is 38.9% in Cumilla. Dhaka (9.5%) has the lowest tonsillitis rate, while Barisal (21.6%) has the highest. Low back discomfort, elbow pain, neck pain, osteoporosis, and osteoarthritis differ by city corporation. Chittagong has the lowest low back pain (34.4%) and Dhaka has the greatest elbow (68.1%) and low back (70.7%). Cumilla (25.3%) has the most osteoporosis, whereas Dhaka (32.8%) has the most osteoarthritis. Mental health indicators like sleeplessness, Industrial Emissions Directive (IED), depression, anxiety, and discontent differ by region. Dhaka has the highest rates of anxiety (81.0%) and sleeplessness (56.0%), while Barisal has the lowest rates of IED (24.6%), insomnia (42.1%), and discontent (37.1%), and Cumilla has the highest depression rate (82.1%). Geographical differences can affect infections and skin problems. Dhaka (40.9%) has the most infections, whereas Chittagong (35.1%) has the most skin diseases. Barisal has often lower infection rates (19.7%).

### 3.2 Multi-level model

**Fig. 2:**
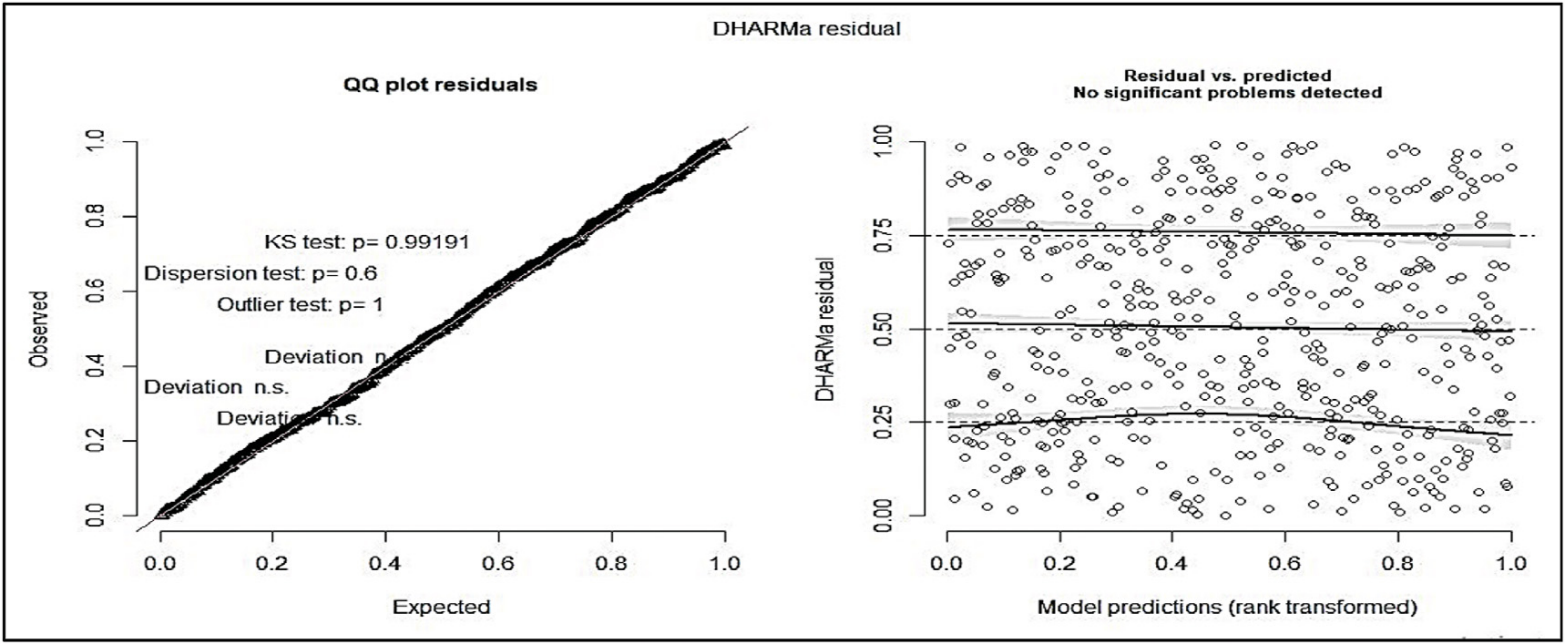
Identification of distributional pattern.

Fig. 2 shows the Q-Q residual plot. This helps to examine the residuals of the multi-level model. The Kolmogorov Smirnov (KS) test pvalue for distributional plot residuals is 0.99, showing a satisfactory fit to the normal distribution assumption. The *p* value strongly suggests that the residual distribution is normal. In the graph above, residuals are randomly distributed about zero with no patterns or trends, suggesting variance homogeneity. Main mixed effect model presumptions include the Dharma residual plot. Additional diagnostics, including the dispersion test (*p* = 0.6) and the outlier test (*p* = 1), further supported that the residuals behaved appropriately. It suggests the null hypothesis of no excess dispersion is accepted. That means model assumptions are met fairly.

These tests compare models with and without that variable to identify each fixed effect predictor’s contribution (Supplementary Table 5). Age, unsafe housing, hygiene, and safety all have *p* values below 0.05, indicating that they significantly affect model likelihood. Furthermore, All variance inflation factor values are less than 2, hence this analysis has no multicollinearity issues. The above correlation matrix indicates the same that there is very weak relationship or no significant correlation among the covariates (Table 1). This maintains model’s fit.

**Table 1:**
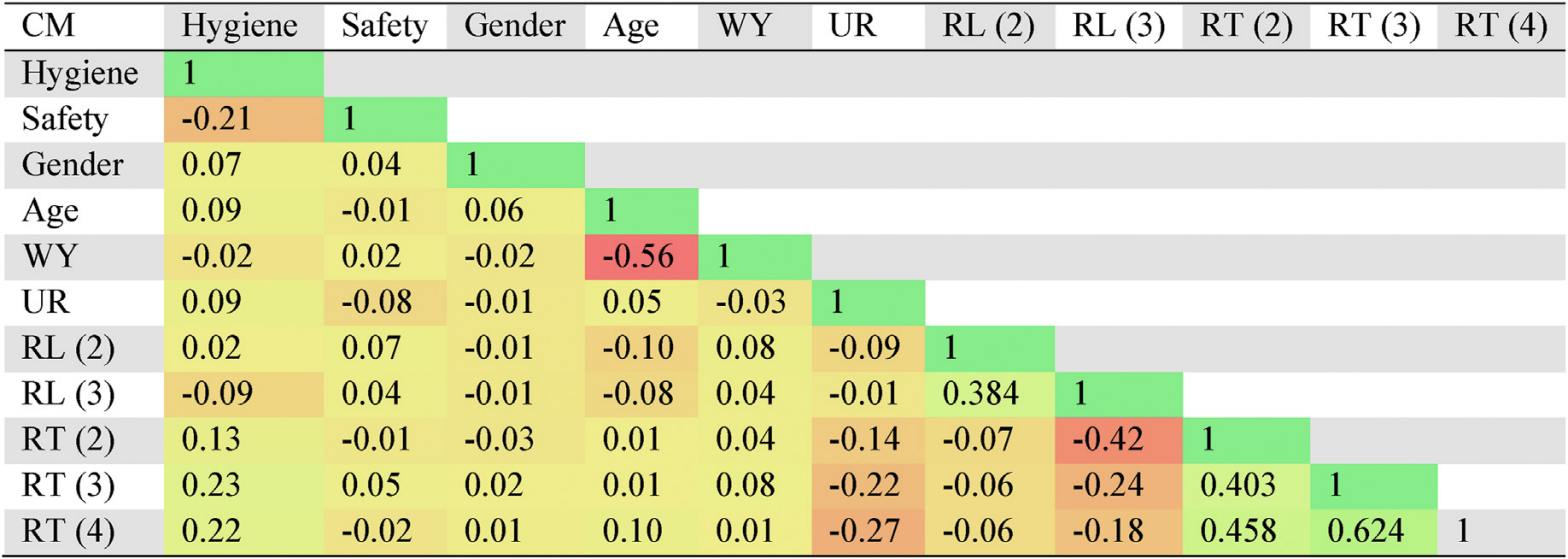
Correlation matrix for respiratory disease

Supplementary Table 6 shows that hygiene, safety, age, and bad housing affect respiratory diseases. Keeping all other factors constant, patients who practice personal cleanliness had about 50% lower log chances of respiratory illness. Maintaining personal safety reduces respiratory illness risk by 46%. One year of age increases respiratory disease risk by 1.024 times. Living in an unhealthy home increases the log odds of getting the disease by 90.5%, holding all other factors constant. Also, the analysis deployed a mixed-effects model with “City Corporations” as the grouping variable for the investigation of diseases and health status variation across regions. In random effect, a moderate amount of variation was found for respiratory diseases (standard deviation = 0.34), indicating unexplained variability among cities after accounting for the fixed effects.

Supplementary Table 7 indicates hygiene, unhealthy living, and geography effect dermatological diseases. Personal hygiene reduces dermatological disease risk by 62%, all else being equal. Keeping all other characteristics equal, living in an unhealthy home increases log odds of the disease by 81.1%. City collectors had a 50% higher risk of dermatological disease than municipal waste collectors. Villagers have a 70% lower chance than city dwellers. Mud hut occupants had 5.4 times the dermatological disease risk of building residents. Keeping all other things constant, sack house probability is 2.88 times the building probability. Dermatological illnesses are consistent throughout city corporations. This means that city corporation personnel have similar issues.

Supplementary Table 8, shows that safety and hygiene have negative coefficients and odds ratios are below 1. This shows that hygiene and safety standards reduce gastrointestinal ailment risk [27]. Odds ratios and confidence intervals demonstrate association direction, size, and precision. Good hygiene or safety behaviors reduce the risk of gastrointestinal disease by 60% and 50%, respectively, compared with bad habits (ORs of 0.40 and 0.50). The OR for age is somewhat over 1, with a small but significant positive coefficient. Age may be a modest risk factor; however, safety and hygiene lessen the probabilities when maintained. The random effect model shows that there is a moderate variation across the nation for gastrointestinal diseases which is seen that most of the respondents have various skin diseases and rash problems.

Workers with excellent cleanliness have about 80% fewer acute irritation symptoms (OR = 0.197). This has the greatest hygienic impact (Supplementary Table 9). Working years have a negative parameter estimate, with odds decreasing by 5% per year. The odds are approximately twice as high in municipal areas as elsewhere. This suggests city living may expose residents to the environment. Small sample sizes limit their power for some categories, but other residential variables, such as house type, are not significant. The random effect model shows no variation for acute irritation problems across the country.

Supplementary Table 10 represents that the random effect for health status is zero indicating health status of the workers across all city corporations is the same. Moreover, hygiene, safety, age, municipal dwelling location, and tin shed residence kinds affect health. Keeping other covariates constant, those who maintain personal hygiene had about 68% lower log odds of poor health. Maintaining personal safety reduces the risk of poor health by 54%. One year of age increases the risk of poor health by 1.053 times. Tin shed dwellers have 49% lower log odds of poor health than building dwellers with all other variables constant. Villagers are 57% less likely to be unhealthy than city dwellers.

**Fig. 3:**
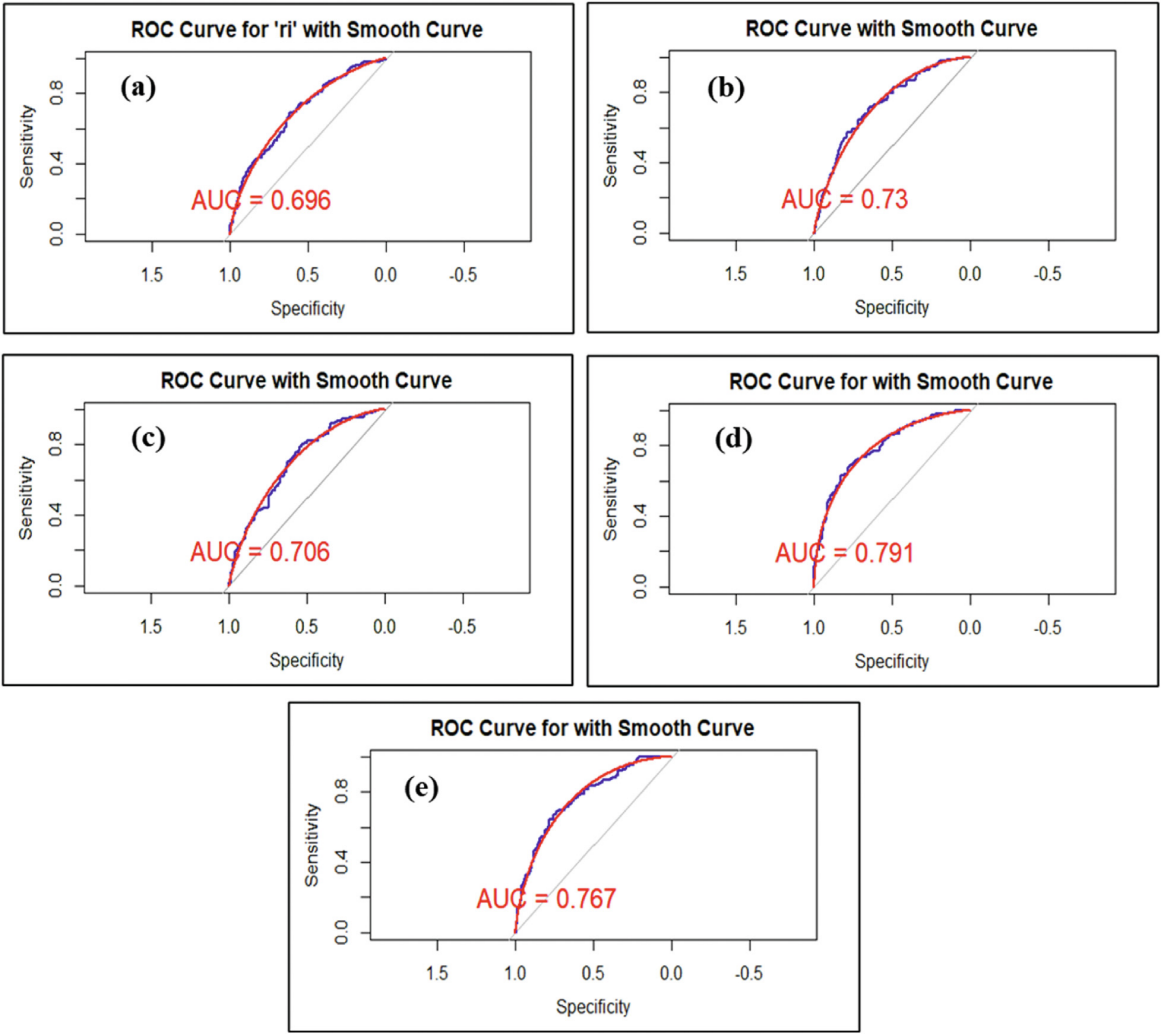
Receiver operating characteristics (ROC) curve for (a) respiratory diseases, (b) dermatological diseases, (c) gastrointestinal diseases, (d) acute irritation symptoms, and (e) health status.

Fig. 3 depicts the model discriminatory power in distinguishing between individuals with and without disease. Models with an AUC (area under the curve) value higher than 0.7 are generally considered to be reliable. AUC for respiratory model is 0.696 (Fig. 3a), which indicates moderate discriminatory power. While this value is slightly below 0.70, it still suggests that the model performs reasonably well. The model AUC for dermatological diseases, gastrointestinal diseases, acute irritation symptoms, and health status are 0.73, 0.706, 0.791, and 0.76, respectively (Fig. 3b–e) indicating that the model’s ability to correct classification is moderately excellent for all, also indicating good discrimination.

## 4 Discussion

The descriptive research shows waste collectors in Bangladesh’s city corporations’ inadequate living conditions, hygiene, safety, and illness burden. It highlights the fact that many people live in hazardous tin dwellings without clean water or sanitation. Nearly 47% of the participants are healthy, while 53% are not [28]. The possible reasons for this imbalance include health risks associated with waste collection methods and inadequate access to healthcare services. Sanitary facilities are available to 58.4% and clean water to 81.3% [29]. Additionally, 65.9% of garbage collectors live in unhealthy housing across all city corporations, ranging from 25% in Dhaka to 84.8% in Khulna [30]. It highlights their poverty: 58.4% lack sanitation, 81.3% lack clean water, and 65.9% live in filthy homes. Most of the housing (55.1%) is tin huts; however, each city has a varied mix. Waste collectors are mostly men (79.6%), while Dhaka has 29.6% female collectors and other cities have 7.1%. The majority (69.2%) live in cities; however, residential regions vary [12,19].

Personal hygiene and safety equipment use are poor [31]. Poor hygiene is common: 41% don't shower after work, 27% don't wash their body after defection, 48% don't have clean nails and around 49% don't have clean clothes. Poor safety: 60% don't use masks, 71% don't wear gloves, and 75% don't have boots. Waste collectors have many respiratory illnesses (19.1% have asthma, 29.7% have cough). Also common are digestive diseases (59.3% stomach pain, 24.1% diarrhea) [32]. Several dermatological, mental, and muscular-skeletal diseases are frequent. Limited access to these resources is a contributor to poor hygiene and health issues. The worrying findings require quick action to improve their welfare.

The multi-level respiratory illness model found that younger age, healthy living conditions, and safety and hygiene measures significantly reduced respiratory disease risk. The disease was more likely in older adults and unhealthy homes [33]. The model accounted for city corporation geographic clustering with a random intercept. Only a fairly efficient classification of healthy and unwell persons was shown by the AUC. The dermatological illness model also shows that hygiene, bad housing, and geography affect dermatological diseases. Keeping all other factors equal, patients who maintain personal cleanliness have 62% lower log chances of dermatological disease. This suggests that proper personal hygiene significantly reduces the risk of skin diseases, likely due to reduced exposure to pathogens and irritants. Living in an unsanitary home increases dermatological disease risk by 81.1%. Village garbage collectors are 70% safer than municipal collectors and 50% safer than city residents. Sack house residents have a 2.88-fold greater risk and mud house residents a 5.4-fold higher risk than building residents. This might be due to lower exposure to hazardous waste and generally improved living conditions in rural areas compared to urban environments. As shown by the table's negative coefficients and odds ratios below 1, superior hygiene and safety practices reduce gastrointestinal disease occurrence. Good hygiene and safety reduce disease by 60% and 50%, respectively. The odds ratio of age is somewhat above 1, indicating a small favorability [34].

Good cleanliness reduces acute discomfort by 80%. The causes of irritation vary [35]. Every additional working year reduces the odds by 5%, showing tolerance. Municipal dwellings show twice the probability, implying environmental exposure. The overall health status model says age, tin shed residence kinds, municipal residence, hygiene, and safety affect their health. Hygiene decreases illness by 68%, while safety reduces it by 54%. Each year of age raises the likelihood 1.053 times. Living in a tin shed reduces the log odds of poor health by 49% compared with living in structures, holding all other characteristics constant. Village life reduces poor health by 57% compared with city life. Lack of cleanliness and protective gear increases the risk of gastrointestinal, musculoskeletal, dermatological, and respiratory problems [11].

Bangladeshi waste collectors live in horrible conditions, lack hygiene and safety equipment, and have high disease rates according to the complete investigation. Many live in unsanitary homes without clean water or sanitation. The data clearly show that collectors’ difficult living and working conditions impair their health.

## 5 Conclusion

The study asserts that inadequate living conditions, insufficient safety protocols, and substandard hygiene practices have a detrimental impact on the waste collectors employed by the Bangladesh’s city corporation. Both univariate and bivariate studies demonstrate a correlation between these traits and gastrointestinal, dermatological, mental, and respiratory diseases. Age, living conditions, and cleanliness are significant predictors of respiratory disorders in multi-level models, whereas personal hygiene, dwelling type, and geography have an impact on dermatological problems. These results provide insights into the geographic distribution of diseases, indicating that illnesses have no significant geographical variability. The results underscore the necessity of enhancing the living and working conditions of waste collectors to safeguard their health.

### 5.1 Recommendation

The study emphasizes the need for integrated actions to address occupational health risks faced by waste collectors, including improving living conditions, enforcing age restrictions, implementing safety protocols, increasing healthcare access, raising awareness, reforming waste collection practices, providing vaccinations, empowering collectors, offering alternate income sources, and extending social security benefits.

### 5.2 Limitations of the study

Some drawbacks of the study include its cross-sectional design, reliance on self-reported data, which is susceptible to bias, inability to prove a causal association, potential presence of confounding variables, and absence of qualitative insight. Furthermore, the survey did not address topics of a sensitive nature, such as drug addiction.

## CRediT authorship contribution statement

**Safayet Hossain:** Writing – original draft, Software, Methodology, Formal analysis. **Md Farhad Hossain:** Validation, Supervision, Methodology, Investigation. **Bowen Liu:** Writing – review & editing, Validation. **Anjuman Ara:** Data curation. **Haneen Alsaoud:** Visualization, Validation. **Md Abdul Majed Patwary:** Supervision.

## Conflicts of interest

The authors whose names are listed immediately below certify that they have no affiliations with or involvement in any organization or entity with any financial interest (such as honoraria; educational grants; participation in speakers' bureaus; membership, employment, consultancies, stock ownership, or other equity interest; and expert testimony or patent-licensing arrangements), or non-financial interest (such as personal or professional relationships, affiliations, knowledge, or beliefs) in the subject matter or materials discussed in this manuscript.

All authors have read the manuscript and agreed for submission with no conflict of interests.
