# Supplementary material for: Health Challenges Among Waste Collectors in Bangladesh: Exploring Risk Factors Using Multi-level Modeling
Source: Saf Health Work. 2024 Nov 14;16(1):13–20. doi: 10.1016/j.shaw.2024.10.007 (PMC11959559; doi:10.1016/j.shaw.2024.10.007)
Supplement: Multimedia component 1 [file mmc1.docx]

**“Health Challenges among Waste Collectors in Bangladesh: Exploring Risk Factors Using Multilevel Modeling**

Shafayet Hossain^1^, MdFarhad Hossain^2, 1, *^, Bowen Liu^2^, Anjuman Ara^1^, Haneen Alsaoud^2^, and Md Abdul Majed Patwary^3^

^1^Department of Statistics, Comilla University, Cumilla 3506, Bangladesh

^2^Department of Mathematics and Statistics, Division of Computing, Analytics and Mathematics, Science and Engineering, University of Missouri, Kansas-City, USA

^3^Department of Chemistry, Comilla University, Cumilla 3506, Bangladesh

***Correspondence:** farhad390ju@gmail.com


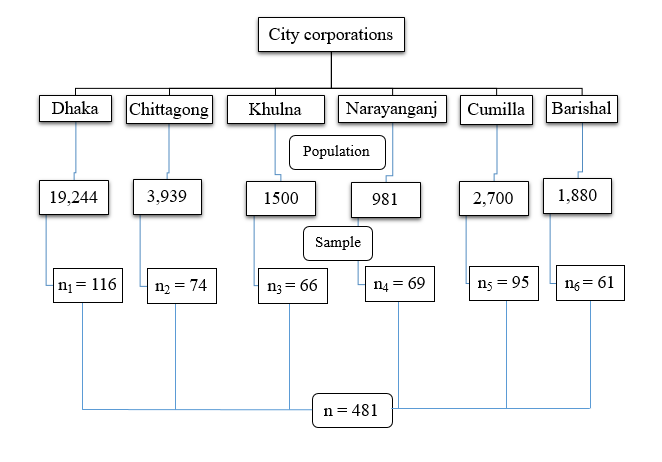


Supplementary Fig. 1: Calculation of sample size for the study


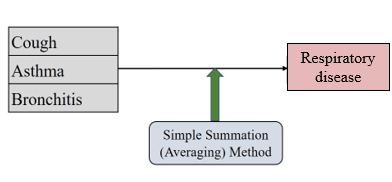


Supplementary Fig. 2: Computation of outcome of interest using averaging method

Supplementary Table 1:Socio-demographic characteristics of the respondents by City Corporations.

|  | | | **City Corporations** | | | | | | **Total** |
| --- | --- | --- | --- | --- | --- | --- | --- | --- | --- |
| **Variables** | **Cate.** |  | **Barisal** | **Chittagong** | **Cumilla** | **Dhaka** | **Khulna** | **Narayan** |  |
| Unhealthy residence | No | Counts (n) | 16 | 14 | 17 | 87 | 10 | 30 | 164 |
|  |  | Within CC (%) | 26.2 | 18.9 | 7.4 | 75.0 | 15.2 | 43.5 | 34.1 |
|  | Yes | Counts (n) | 45 | 60 | 78 | 29 | 56 | 39 | 317 |
|  |  | Within CC (%) | 73.8 | 81.1 | 82.1 | 25.0 | 84.8 | 56.5 | 65.9 |
| Gender | Female | Counts (n) | 7 | 7 | 6 | 29 | 26 | 23 | 98 |
|  |  | Within CC (%) | 11.5 | 9.5 | 6.3 | 25.0 | 39.4 | 33.3 | 20.4 |
|  | Male | Counts (n) | 54 | 67 | 89 | 87 | 40 | 46 | 383 |
|  |  | Within CC (%) | 88.5 | 90.5 | 93.7 | 75.0 | 60.6 | 66.7 | 79.6 |
| Residence type | Building | Counts (n) | 3 | 9 | 11 | 108 | 1 | 20 | 152 |
|  |  | Within CC (%) | 4.9 | 12.2 | 11.6 | 93.1 | 1.5 | 29.0 | 31.6 |
|  | Mud House | Counts (n) | 0 | 4 | 13 | 0 | 1 | 0 | 18 |
|  |  | Within CC (%) | 0.0 | 5.4 | 13.7 | 0.0 | 1.5 | 0.0 | 3.7 |
|  | Sack House | Counts (n) | 11 | 14 | 17 | 0 | 4 | 0 | 46 |
|  |  | Within CC (%) | 18.0 | 18.9 | 17.9 | 0.0 | 6.1 | 0.0 | 9.6 |
|  | Tin Shed | Counts (n) | 47 | 47 | 54 | 8 | 60 | 49 | 265 |
|  |  | Within CC (%) | 77.0 | 63.5 | 56.8 | 6.9 | 90.9 | 71.0 | 55.1 |
| Residence  location | City | Counts (n) | 21 | 33 | 30 | 116 | 65 | 68 | 333 |
|  |  | Within CC (%) | 34.4 | 44.6 | 31.6 | 100 | 98.5 | 98.6 | 69.2 |
|  | Municipal | Counts (n) | 11 | 13 | 35 | 0 | 0 | 0 | 59 |
|  |  | Within CC (%) | 18.0 | 17.6 | 36.8 | 0.0 | 0.0 | 0.0 | 12.3 |
|  | Village | Counts (n) | 29 | 28 | 30 | 0 | 1 | 1 | 89 |
|  |  | Within CC (%) | 47.5 | 37.8 | 31.6 | 0.0 | 1.5 | 1.4 | 18.5 |
| Sanitation  facility | No | Counts (n) | 44 | 59 | 73 | 26 | 54 | 25 | 281 |
|  |  | Within CC (%) | 72.1 | 79.7 | 76.8 | 22.4 | 81.8 | 36.2 | 58.4 |
|  | Yes | Counts (n) | 17 | 15 | 22 | 90 | 12 | 44 | 200 |
|  |  | Within CC (%) | 27.9 | 20.3 | 23.2 | 77.6 | 18.2 | 63.8 | 41.6 |
| Clean  Water | No | Counts (n) | 46 | 58 | 88 | 95 | 59 | 45 | 391 |
|  |  | Within CC (%) | 75.4 | 78.4 | 92.6 | 81.9 | 89.4 | 65.2 | 81.3 |
|  | Yes | Counts (n) | 15 | 16 | 7 | 21 | 7 | 24 | 90 |
|  |  | Within CC (%) | 24.6 | 21.6 | 7.4 | 18.1 | 10.6 | 34.8 | 18.7 |

[CC*= City Corporations]

Supplementary Table 2:Hygiene practices maintained by the waste collectors.

| **Personal Hygiene** | **Categories** | **Percentage, %** |
| --- | --- | --- |
| Take a bath after work | yes | 59.3 |
|  | no | 40.7 |
| Take a bath after defection | yes | 73.4 |
|  | no | 26.6 |
| Soap bath | yes | 67.6 |
|  | no | 32.4 |
| Clean nail | yes | 52.2 |
|  | no | 47.8 |
| Clean hair | yes | 53.2 |
|  | no | 46.8 |
| Clean cloth | yes | 51.1 |
|  | no | 48.9 |

Supplementary Table 3:Safety materials used by the waste collectors.

| **Safety materials** | **Response** | **Percentage, %** |
| --- | --- | --- |
| Mask | yes | 40.5 |
|  | no | 59.5 |
| Gloves | yes | 29.1 |
|  | no | 70.9 |
| Boot | yes | 25.4 |
|  | no | 74.6 |
| Apron | yes | 48.2 |
|  | no | 51.8 |

Supplementary Table 4:Disease comparison by region, showing the prevalence of various diseases among waste collectors in City Corporations.

|  | | **City Corporations,** % | | | | | |  |
| --- | --- | --- | --- | --- | --- | --- | --- | --- |
| **Variables** | **Cate.** | **Barisal** | **Chittagong** | **Cumilla** | **Dhaka** | **Khulna** | **Narayan.** | **Total** |
| Cough | Yes | 16.4 | 47.3 | 28.4 | 37.1 | 18.2 | 23.2 | 29.7 |
|  | No | 83.6 | 52.7 | 71.6 | 62.9 | 81.8 | 76.8 | 70.3 |
| Asthma | Yes | 16.4 | 25.7 | 22.1 | 15.5 | 31.8 | 4.3 | 19.1 |
|  | No | 83.6 | 74.3 | 77.9 | 84.5 | 68.2 | 95.7 | 80.9 |
| Bronchitis | Yes | 36.1 | 18.9 | 12.6 | 14.7 | 12.1 | 4.3 | 15.8 |
|  | No | 63.9 | 81.1 | 87.4 | 85.3 | 87.9 | 95.7 | 84.2 |
| Nausea | Yes | 23.0 | 27.0 | 31.6 | 27.6 | 48.5 | 10.1 | 28.1 |
|  | No | 77.0 | 73.0 | 68.4 | 72.4 | 51.5 | 89.9 | 71.9 |
| Gastric | Yes | 50.8 | 52.7 | 45.3 | 77.6 | 62.1 | 59.4 | 59.3 |
|  | No | 49.2 | 47.3 | 54.7 | 22.4 | 37.9 | 40.6 | 40.7 |
| Diarrhea | Yes | 9.8 | 36.5 | 29.5 | 26.7 | 21.2 | 14.5 | 24.1 |
|  | No | 90.2 | 63.5 | 70.5 | 73.3 | 78.8 | 85.5 | 75.9 |
| Anorexia | Yes | 16.4 | 28.4 | 32.6 | 35.3 | 43.9 | 17.4 | 29.9 |
|  | No | 83.6 | 71.6 | 67.4 | 64.7 | 56.1 | 82.6 | 70.1 |
| Eye problem | Yes | 26.2 | 9.5 | 17.9 | 46.6 | 19.7 | 18.8 | 24.9 |
|  | No | 73.8 | 90.5 | 82.1 | 53.4 | 80.3 | 81.2 | 75.1 |
| Hyposmia | Yes | 27.9 | 25.7 | 38.9 | 20.7 | 31.8 | 15.9 | 26.8 |
|  | No | 72.1 | 74.3 | 61.1 | 79.3 | 68.2 | 84.1 | 73.2 |
| Tonsilitis | Yes | 9.8 | 21.6 | 14.7 | 9.5 | 18.2 | 5.8 | 13.1 |
|  | No | 90.2 | 78.4 | 85.3 | 90.5 | 81.8 | 94.2 | 86.9 |
| Hypogeusia | Yes | 29.5 | 37.8 | 73.7 | 32.8 | 28.8 | 17.4 | 38.5 |
|  | No | 70.5 | 62.2 | 26.3 | 67.2 | 71.2 | 82.6 | 61.5 |
| Low back pain | Yes | 34.4 | 36.5 | 47.4 | 70.7 | 48.5 | 73.9 | 53.6 |
|  | No | 65.6 | 63.5 | 52.6 | 29.3 | 51.5 | 26.1 | 46.4 |
| Elbow pain | Yes | 29.5 | 31.1 | 40.0 | 68.1 | 45.5 | 65.2 | 48.4 |
|  | No | 70.5 | 68.9 | 60.0 | 31.9 | 54.5 | 34.8 | 51.6 |
| neck pain | Yes | 50.8 | 36.5 | 56.8 | 66.4 | 45.5 | 63.8 | 54.7 |
|  | No | 49.2 | 63.5 | 43.2 | 33.6 | 54.5 | 36.2 | 45.3 |
| Osteoporosis | Yes | 11.5 | 14.9 | 25.3 | 21.6 | 21.2 | 17.4 | 19.3 |
|  | No | 88.5 | 85.1 | 74.7 | 78.4 | 78.8 | 82.6 | 80.7 |
| Osteoarthritis | Yes | 6.6 | 5.4 | 9.5 | 32.8 | 10.6 | 20.3 | 15.8 |
|  | No | 93.4 | 94.6 | 90.5 | 67.2 | 89.4 | 79.7 | 84.2 |
| Insomnia | Yes | 42.6 | 51.4 | 42.1 | 56.0 | 57.6 | 47.8 | 49.9 |
|  | No | 57.4 | 48.6 | 57.9 | 44.0 | 42.4 | 52.2 | 50.1 |
| IED | Yes | 24.6 | 16.2 | 28.4 | 75.9 | 42.4 | 58.0 | 43.7 |
|  | No | 75.4 | 83.8 | 71.6 | 24.1 | 57.6 | 42.0 | 56.3 |
| Anxiety | Yes | 62.3 | 41.9 | 36.8 | 81.0 | 59.1 | 60.9 | 58.0 |
|  | No | 37.7 | 58.1 | 63.2 | 19.0 | 40.9 | 39.1 | 42.0 |
| Depression | Yes | 55.7 | 58.1 | 82.1 | 83.6 | 62.1 | 46.4 | 67.6 |
|  | No | 44.3 | 41.9 | 17.9 | 16.4 | 37.9 | 53.6 | 32.4 |
| Dissatisfaction | Yes | 63.9 | 48.6 | 67.4 | 37.1 | 66.7 | 73.9 | 57.6 |
|  | No | 36.1 | 51.4 | 32.6 | 62.9 | 33.3 | 26.1 | 42.4 |
| Skin Diseases | Yes | 21.3 | 35.1 | 30.5 | 21.6 | 27.3 | 21.7 | 26.2 |
|  | No | 78.7 | 64.9 | 69.5 | 78.4 | 72.7 | 78.3 | 73.8 |
| Infection | Yes | 19.7 | 31.1 | 30.5 | 16.4 | 40.9 | 13.0 | 24.7 |
|  | No | 80.3 | 68.9 | 69.5 | 83.6 | 59.1 | 87.0 | 75.3 |

Supplementary Table 5:Likelihood ratio tests for fixed effects, assessing the significance of various covariates in the multilevel model.

| Hygiene | 9.1385 | 0.002503 ** | 1.183363 |
| --- | --- | --- | --- |
| Safety | 8.3439 | 0.003870 ** | 1.077946 |
| Gender | 0.1494 | 0.699144 | 1.015685 |
| Age | 6.0925 | 0.013575 * | 1.521311 |
| Working year | 1.1034 | 0.293530 | 1.489459 |
| Unhealthy Residence | 6.8484 | 0.008872 ** | 1.143873 |
| Residence Location | 3.1132 | 0.210851 | 1.306631 |
| Residence Types | 0.4826 | 0.922707 | 1.549094 |

Supplementary Table 6:Result for Multilevel Linear Mixed Models for Respiratory Diseases

| **Fixed Effects** | | | | | | | **Random effects** |
| --- | --- | --- | --- | --- | --- | --- | --- |
| **Predictors** | **B** | **Std. Error** | **P-value** | **AOR** | **95% CI.** | | **Std.**  **Dev.** |
|  |  |  |  |  | **Lower** | **Upper** |  |
| Hygiene (Yes) | -0.699 | 0.231 | 0.002 ** | 0.496 | 0.043 | 0.949 | 0.34 |
| Safety (Yes) | -0.624 | 0.218 | 0.004 ** | 0.535 | 0.108 | 0.962 |  |
| Gender (Female) | 0.096 | 0.249 | 0.698 | 1.101 | 0.613 | 1.589 |  |
| Age | 0.024 | 0.010 | 0.015 * | 1.024 | 1.004 | 1.044 |  |
| Working year | -0.013 | 0.012 | 0.297 | 0.986 | 0.962 | 1.010 |  |
| Unhealthy Residence (Yes) | 0.645 | 0.248 | 0.009 ** | 1.905 | 1.419 | 2.391 |  |
| Residence Location (Municipal) | -0.510 | 0.346 | 0.134 | 0.595 | 0.083 | 1.273 |  |
| Residence Location (Village) | -0.461 | 0.340 | 0.176 | 0.630 | 0.036 | 1.296 |  |
| Residence Types (Mud House) | -0.046 | 0.647 | 0.942 | 0.954 | 0.314 | 2.222 |  |
| Residence Types (Sack House) | -0.257 | 0.449 | 0.565 | 0.772 | 0.108 | 1.652 |  |
| Residence Types (Tin Shed) | -0.179 | 0.310 | 0.562 | 0.835 | 0.227 | 1.443 |  |
| Intercept | -0.47 | 0.537 | 0.380 | -- | -- | -- |  |

Supplementary Table 7:Result for Multilevel Linear Mixed Models for Dermatological Diseases

| **Fixed Effects** | | | | | | | **Random Effects** |
| --- | --- | --- | --- | --- | --- | --- | --- |
| **Predictors** | **B** | **Std. Error** | **P-value** | **AOR** | **95% CI.** | | **Std.**  **Dev.** |
|  |  |  |  |  | **Lower** | **Upper** |  |
| Hygiene (Yes) | -0.959 | 0.235 | 0.00*** | 0.383 | 0.077 | 0.843 | 0 |
| Safety (Yes) | -0.324 | 0.222 | 0.1456 | 0.723 | 0.287 | 1.159 |  |
| Gender (Female) | 0.451 | 0.268 | 0.0926 | 1.569 | 1.043 | 2.095 |  |
| Age | 0.014 | 0.010 | 0.1555 | 1.014 | 0.994 | 1.034 |  |
| Working year | -0.008 | 0.013 | 0.5453 | 0.992 | 0.966 | 1.018 |  |
| Unhealthy Residence (Yes) | 0.595 | 0.262 | 0.0232* | 1.813 | 1.299 | 2.327 |  |
| Residence Location (Municipal) | -0.685 | 0.331 | 0.0384* | 0.504 | 0.144 | 1.152 |  |
| Residence Location (Village) | -1.178 | 0.351 | 0.000*** | 0.307 | 0.381 | 0.995 |  |
| Residence Types (Mud House) | 1.687 | 0.645 | 0.0089** | 5.401 | 4.137 | 6.665 |  |
| Residence Types (Sack House) | 1.059 | 0.442 | 0.0165* | 2.884 | 2.018 | 3.750 |  |
| Residence Types (Tin Shed) | 0.110 | 0.281 | 0.6951 | 1.11 | 0.558 | 1.662 |  |
| Intercept | -1.120 | 0.512 | 0.0286* | -- | -- | -- |  |

Supplementary Table 8:Result for Multilevel Linear Mixed Models for Gastrointestinal Diseases

| **Fixed Effects** | | | | | | | **Random Effects** |
| --- | --- | --- | --- | --- | --- | --- | --- |
| **Predictors** | **B** | **Std. Error** | **P-value** | **AOR** | **95% CI.** | | **Std.**  **Dev.** |
|  |  |  |  |  | **Lower** | **Upper** |  |
| Hygiene (Yes) | -0.902 | 0.253 | 0.0004*** | 0.405 | 0.299 | 1.472 |  |
| Safety (Yes) | -0.689 | 0.220 | 0.0017** | 0.502 | 0.006 | 0.998 |  |
| Gender (Female) | -0.449 | 0.252 | 0.0746 | 0.638 | 0.207 | 1.068 |  |
| Age | 0.021 | 0.010 | 0.0395* | 1.021 | 0.527 | 1.519 |  |
| Working year | -0.003 | 0.013 | 0.7829 | 0.996 | 0.976 | 1.016 |  |
| Unhealthy Residence (Yes) | -0.424 | 0.253 | 0.0936 | 0.654 | 0.629 | 0.678 | 0.27 |
| Residence Location (Municipal) | -0.308 | 0.347 | 0.3740 | 0.734 | 0.238 | 1.229 |  |
| Residence Location (Village) | 0.081 | 0.326 | 0.8041 | 1.084 | 0.403 | 1.761 |  |
| Residence Types (Mud House) | -0.105 | 0.633 | 0.8686 | 0.90 | 0.262 | 1.539 |  |
| Residence Types (Sack House) | -0.028 | 0.445 | 0.9499 | 0.972 | 0.267 | 2.211 |  |
| Residence Types (Tin Shed) | -0.359 | 0.304 | 0.2373 | 0.698 | 0.173 | 1.569 |  |
| Intercept | 0.505 | 0.545 | 0.000366 | -- | -- | -- |  |

Supplementary Table 9:Result for Multilevel Linear Mixed Models for Acute Irritation Symptoms

| **Fixed Effects** | | | | | | | **Random Effects** |
| --- | --- | --- | --- | --- | --- | --- | --- |
| **Predictors** | **B** | **Std. Error** | **P-value** | **AOR** | **95% CI.** | | **Std. Dev.** |
|  |  |  |  |  | **Lower** | **Upper** |  |
| Hygiene (Yes) | -1.62 | 0.279 | 0.000*** | 0.197 | 0.123 | 0.744 |  |
| Safety (Yes) | -0.46 | 0.251 | 0.062 | 0.626 | 0.134 | 1.118 |  |
| Gender (Female) | -0.43 | 0.284 | 0.122 | 0.645 | 0.089 | 1.201 |  |
| Age | 0.06 | 0.012 | 0.000*** | 1.066 | 1.043 | 1.089 |  |
| Working year | -0.04 | 0.016 | 0.002** | 0.953 | 0.922 | 0.984 |  |
| Unhealthy Residence (Yes) | 0.30 | 0.293 | 0.306 | 1.350 | 0.775 | 1.925 | 0 |
| Residence Location (Municipal) | 0.69 | 0.345 | 0.043* | 2.009 | 1.333 | 2.685 |  |
| Residence Location (Village) | 0.06 | 0.374 | 0.866 | 1.065 | 0.332 | 1.798 |  |
| Residence Types (Mud House) | 0.18 | 0.695 | 0.785 | 1.208 | 0.154 | 2.570 |  |
| Residence Types (Sack House) | 0.04 | 0.475 | 0.928 | 1.043 | 0.113 | 1.973 |  |
| Residence Types (Tin Shed) | -0.44 | 0.315 | 0.156 | 0.639 | 0.022 | 1.256 |  |
| Intercept | -1.57 | 0.54 | 0.000 | -- | -- | -- |  |

Supplementary Table 10:Result for Multilevel Linear Mixed Models for Health Status

| **Fixed Effects** | | | | | | | **Random Effects** |
| --- | --- | --- | --- | --- | --- | --- | --- |
| **Predictors** | **B** | **Std. Error** | **P-value** | **AOR** | **95% CI.** | | **Std. Dev.** |
|  |  |  |  |  | **Lower** | **Upper** |  |
| Hygiene (Yes) | -1.155 | 0.242 | 0.000*** | 0.315 | 0.160 | 0.790 |  |
| Safety (Yes) | -0.777 | 0.218 | 0.004*** | 0.460 | 0.032 | 0.888 |  |
| Gender (Female) | -0.172 | 0.254 | 0.4977 | 0.842 | 0.344 | 1.339 |  |
| Age | 0.052 | 0.011 | 0.000*** | 1.053 | 1.032 | 1.075 |  |
| Working year | -0.009 | 0.013 | 0.4821 | 0.991 | 0.965 | 1.017 |  |
| Unhealthy Residence (Yes) | 0.495 | 0.264 | 0.0603 | 1.641 | 1.124 | 2.157 | 0 |
| Residence Location (Municipal) | -0.575 | 0.342 | 0.0928 | 0.563 | 0.107 | 1.233 |  |
| Residence Location (Village) | -0.696 | 0.344 | 0.0431* | 0.498 | 0.176 | 1.173 |  |
| Residence Types (Mud House) | 0.395 | 0.698 | 0.5715 | 1.484 | 0.116 | 2.852 |  |
| Residence Types (Sack House) | 0.424 | 0.473 | 0.3704 | 1.528 | 0.600 | 2.456 |  |
| Residence Types (Tin Shed) | -0.723 | 0.288 | 0.0120* | 0.485 | 0.079 | 1.050 |  |
| Intercept | -1.155 | 0.242 | -- | -- | -- | -- |  |

Supplementary Table 11: Variables included in the indices used for statistical analysis.

| **Variables** | **Categories** | **Indices** |
| --- | --- | --- |
| Gloves | (Yes, No) | Personal Safety |
| Boot | (Yes, No) |  |
| Apron | (Yes, No) |  |
| Mask | (Yes, No) |  |
| Clean cloth | (Yes, No) | Personal Hygiene |
| Clean hair | (Yes, No) |  |
| Clean nail | (Yes, No) |  |
| Soap bath | (Yes, No) |  |
| Soap hand wash after defection | (Yes, No) |  |
| Soap hand wash after work | (Yes, No) |  |
| Infection | (Yes, No) | Dermatological disease |
| Bronchitis | (Yes, No) |  |
| Skin diseases | (Yes, No) |  |
| Dissatisfaction | (Yes, No) | Mental health issues |
| Depression | (Yes, No) |  |
| Anxiety | (Yes, No) |  |
| IED | (Yes, No) |  |
| Insomnia | (Yes, No) |  |
| Osteoarthitis | (Yes, No) | Musculoskeletal diseases |
| Osteoperosis | (Yes, No) |  |
| Neck pain | (Yes, No) |  |
| Elbow pain | (Yes, No) |  |
| Low back pain | (Yes, No) |  |
| Gastric | (Yes, No) | Gastrointestinal diseases |
| Diarrhoea | (Yes, No) |  |
| Nausea | (Yes, No) |  |
| Anorexia | (Yes, No) |  |
| Eyeproblem | (Yes, No) | Acute irritation problems |
| Hypogeusia | (Yes, No) |  |
| Tonsilitis | (Yes, No) |  |
| Hyposmia | (Yes, No) |  |
| Asthma | (Yes, No) | Respiratory diseases |
| Cough | (Yes, No) |  |
| Bronchitis | (Yes, No) |  |
